# Hemodynamically stable oliguric patients usually do not respond to fluid challenge

**DOI:** 10.5935/0103-507X.20200094

**Published:** 2020

**Authors:** Vinicius Brenner Felice, Thiago Costa Lisboa, Lucas Vieira de Souza, Luana Canevese Sell, Gilberto Friedman

**Affiliations:** 1 Central Intensive Care Unit, Complexo Hospitalar Santa Casa de Porto Alegre - Porto Alegre (RS), Brazil.; 2 Postgraduate Program in Pneumological Sciences, Universidade Federal do Rio Grande do Sul - Porto Alegre (RS), Brazil.; 3 Intensive Care Unit, Hospital de Clínicas de Porto Alegre, Faculdade de Medicina, Universidade Federal do Rio Grande do Sul - Porto Alegre (RS), Brazil.; 4 Research and Inovation Institutional Network in Intensive Care, Complexo Hospitalar Santa Casa de Porto Alegre - Porto Alegre (RS), Brazil.

**Keywords:** Oliguria, Fluid therapy/methods, Acute kidney injury, Intensive care units, Oligúria, Hidratação/métodos, Insuficiência renal aguda, Unidades de terapia intensiva

## Abstract

**Objective:**

To evaluate renal responsiveness in oliguric critically ill patients after a fluid challenge.

**Methods:**

We conducted a prospective observational study in one university intensive care unit. Patients with urine output < 0.5mL/kg/h for 3 hours with a mean arterial pressure > 60mmHg received a fluid challenge. We examined renal fluid responsiveness (defined as urine output > 0.5mL/kg/h for 3 hours) after fluid challenge.

**Results:**

Forty-two patients (age 67 ± 13 years; APACHE II score 16 ± 6) were evaluated. Patient characteristics were similar between renal responders and renal nonresponders. Thirteen patients (31%) were renal responders. Hemodynamic or perfusion parameters were not different between those who did and those who did not increase urine output before the fluid challenge. The areas under the receiver operating characteristic curves were calculated for mean arterial pressure, heart rate, creatinine, urea, creatinine clearance, urea/creatinine ratio and lactate before the fluid challenge. None of these parameters were sensitive or specific enough to predict reversal of oliguria.

**Conclusion:**

After achieving hemodynamic stability, oliguric patients did not increase urine output after a fluid challenge. Systemic hemodynamic, perfusion or renal parameters were weak predictors of urine responsiveness. Our results suggest that volume replacement to correct oliguria in patients without obvious hypovolemia should be done with caution.

## Abstract (pt)

**Objetivo:**

Avaliar a responsividade renal após desafio hídrico em pacientes oligúricos na unidade de terapia intensiva.

**Método:**

Conduzimos um estudo observacional prospectivo em uma unidade de terapia intensiva universitária. Pacientes com débito urinário inferior a 0,5mL/kg/hora por 3 horas, com pressão arterial média acima de 60mmHg receberam um desafio hídrico. Examinamos a responsividade renal aos fluidos (definida como débito urinário acima de 0,5mL/kg/hora por 3 horas) após o desafio hídrico.

**Resultados:**

Avaliaram-se 42 pacientes (idade 67 ± 13 anos; APACHE II 16 ± 6). As características dos pacientes foram similares entre os respondedores e os não respondedores renais. Treze pacientes (31%) foram respondedores renais. Antes do desafio hídrico, os parâmetros hemodinâmicos e de perfusão não foram diferentes entre os pacientes que apresentaram aumento do débito urinário e os que não apresentaram. Calcularam-se as áreas sob a curva *receiver operating characteristic* para os níveis pré-desafio hídrico de pressão arterial média, frequência cardíaca, creatinina, ureia, depuração de creatinina, proporção ureia/creatinina e lactato. Nenhum desses parâmetros foi sensível ou suficientemente específico para predizer a reversão da oligúria.

**Conclusão:**

Após obtenção de estabilidade hemodinâmica, os pacientes oligúricos não alcançaram aumento do débito urinário em resposta ao desafio hídrico. Os parâmetros de hemodinâmica sistêmica, perfusão ou renais foram preditores fracos de responsividade urinária. Nossos resultados sugerem que a reposição de volume com objetivo de corrigir oligúria em pacientes sem hipovolemia óbvia deve ser realizada com cautela.

## INTRODUCTION

Hypovolemia is a major risk factor for the development of acute kidney injury (AKI) and is associated with low urinary output.^(1)^ Oliguria is often viewed as a sign of renal hypoperfusion and triggers interventions, such as a fluid challenge, with the aim of improving systemic hemodynamics to improve renal perfusion and consequently renal function.^(2)^ However, oliguria does not always indicate ongoing renal hypoperfusion in a critically ill patient. The pathophysiologic mechanisms implicated in acute oliguria are multifactorial: reversible renal hypoperfusion due to low cardiac output or due to vasoplegic hypotension; augmented liberation of antidiuretic hormone unrelated to kidney perfusion or damage and with maintained glomerular filtration rate, and renal damage.^(3)^ Both experimental and clinical studies showed that the glomerular filtration rate and renal blood flow can be dissociated.^(4-6)^

Thus, there are many uncertainties about when AKI is considered volume responsive, in particular in cases that hypovolemia is unlikely and AKI is not caused by renal hypoperfusion.^(1,7-9)^ In these situations, injudicious use of fluids carries its own risks of contributing to the development or worsening of AKI by fluid overload.^(10,11)^ Nevertheless, hypotension, hypernatremia or low urine sodium concentration have long been considered markers of a low intravascular volume state in an oliguric patient and often leads to a fluid challenge or aggressive fluid repositions.^(12,13)^

As a result, at the bedside, renal responsiveness (i.e., urine output) is observed in only half of oliguric patients, even if there are reasons to consider that the patient's blood volume is depleted.^(12,13)^

Therefore, the objective of this study was to evaluate renal responsiveness in oliguric intensive care unit (ICU) patients after a fluid challenge.

## METHODS

Because this study was observational and did not change daily practice, the Institutional Review Board of the *Complexo Hospitalar Santa Casa de Porto Alegre* approved the present study and waived the requirement for obtaining consent.

This study was performed in the central ICU of the *Complexo Hospitalar Santa Casa de Porto Alegre*, a 20-bed adult medical-surgical ICU. Patients were included if they met the following criteria:^(12)^ oliguria, defined by urine output < 0.5mL/kg/h for 3 consecutive hours; administration of a fluid challenge (500mL of isotonic crystalloids over 15 - 30 minutes) as indicated by the physician in charge and mean arterial pressure (MAP) ≥ 60mmHg.

The indications for fluid challenge were obtained from the physicians in charge or from the patient's records. The exclusion criteria were patients under 18 years old, treated with diuretics on the study day, with stage 3 AKI, pregnancy, with chronic renal failure, with a decision to withhold or withdraw treatment, and likely to die in the next 48 hours.

The main endpoint of the study was renal fluid responsiveness, defined as a post-fluid challenge urine output > 0.5mL/kg/h for more than 3 hours (fluid renal responders -RR).^(12)^ Patient characteristics, Acute Physiology Chronic Health Evaluation II (APACHE II) score at admission,^(14)^ and reasons for ICU admission were recorded. At inclusion (i.e., at the time of oliguria diagnosis; urine output < 0.5mL/kg/hours for 3 consecutive hours) and at completion of the fluid challenge, we recorded MAP, heart rate (HR) and norepinephrine infusion rate. Capillary refill time both at inclusion and after the fluid challenge was recorded as normal (< 5 seconds) or abnormal (≥ 5 seconds).^(15)^ All available routine laboratory measurements of study interest during the previous 3 hours were recorded (serum creatinine, blood urea, arterial lactate, central venous and arterial blood gases). The same parameters were recorded in the following 3 hours after the fluid challenge if available. Creatinine clearance was calculated using the Cockcroft-Gault equation.

### Statistical analysis

Categorical variables were compared using a Chi-squared test. The area under the Receiver Operating Characteristic (AUROC) Curve to predict urine responsiveness was built for MAP, HR, serum creatinine, blood urea, urea/creatinine ratio, creatinine clearance and arterial lactate. We determined the optimal threshold value for each variable. All analyses were performed using IBM Statistical Package for Social Science (SPSS) Statistics software (IBM, Armonk, NY, USA). All p values were two-tailed, and a p value < 0.05 was considered significant. Values are expressed as number and percentage, mean or median and interquartile range accordingly.

## RESULTS

Forty-two patients were evaluated between March 2017 and October 2017. The patient features are shown in table 1. The most frequent reasons for a fluid challenge were a lactate level > 3mmol/L (n = 12), elevated serum creatinine and/or urea (n = 19) and norepinephrine dose infusion > 0.20µg/kg/hour, without differences between groups. Dynamic fluid responsiveness was tested in only 2 patients before the fluid challenge.

**Table 1:** Patients characteristics

Characteristic	All patients (n = 42)	Renal responders (n = 13)	Renal nonresponders (n = 29)	p value
Age (years)	67 ± 13	63 ± 12	68 ± 13	0.22
Sex male	24 (57)	8 (61)	16 (55)	0.96
Comorbidities				
Diabetes mellitus	12	4	8	1.00
Hypertension	26	7	19	0.51
Coronary disease	7	1	6	0.41
Cancer	19	7	12	0.68
Other	4	1	3	NA
Organ failure				
APACHE II	16 ± 6	16 ± 7	16 ± 6	0.98
Mechanical ventilation	23	5	18	0.28
PaO_2_/FiO_2_	296 (207 - 352)	397 (325 - 427)	259 (202 - 327)	0.02
Platelet count, 10^3^/L	212 (173 - 267)	210 (174 - 247)	215 (166 - 280)	0.99
Hemoglobin (g/dL)	11 ± 3	11 ± 5	10 ± 2	0.46
Bicarbonate (mmol/L)	21 ± 6	20 ± 4	21 ± 6	0.37
Reasons for ICU admission				
Sepsis	17	6	11	0.99
Elective surgery	12	5	7	0.51
Emergency surgery	10	2	8	0.70
Others	3	1	2	NA

NA - not available; APACHE II - Acute Physiology and Chronic Health Evaluation II score; PaO_2_/FiO_2_ - ratio of arterial oxygen tension to inspired oxygen fraction; ICU - intensive care unit. Results expressed as mean ± standard deviation and median (25% - 75% confidence interval).

Table 2 shows all the parameter changes before and after the fluid challenge. Urine output increased after the fluid challenge. The HR and MAP before and after fluid challenge were similar. Blood lactate levels (n = 37) and norepinephrine infusion rates (n = 26) decreased after the fluid challenge. Arterial and central venous blood gases were measured in 22 patients, but neither central venous oxygen saturation (SvcO_2_) nor central venous to arterial carbon dioxide difference (Pcv-aCO_2_) improved after the volume load.

**Table 2:** Hemodynamic, perfusion and renal parameters in renal responders and renal nonresponders

Variables	Renal responders (n = 13)		Renal nonresponders (n = 29)		All patients (n = 42)	
	Pre	Pos	Pre	Pos	Pre	Pos
Diuresis (mL/kg/hour)	0.22 ± 0.15	1.09 ± 0.49*	0.19 ± 0.16	0.26 ± 0.14†	0.21 ± 0.16	0.49 ± 0.5*
HR (beats/minute)	95 ± 22	93 ± 17	94 ± 22	96 ± 23	95 ± 27	95 ± 21
MAP (mmHg)	75 ± 13	80 ± 11	80 ± 16	81 ± 16	78 ± 15	80 ± 14
CRT (normal/abnormal)	12/1	13/0	23/3	24/2	35/4	37/2
Serum creatinine (mg/dL)	1.7 ± 1.0		1.4 ± 0.6		1.5 ± 0.8	
Blood urea (mmol/L)	80 ± 39		70 ± 44		73 ± 42	
Urea/creatinine	57 ± 38		53 ± 24		54 ± 28	
Creatinine clearance	53 ± 28		58 ± 41		58 ± 37	
Arterial lactate (mmol/L)	2.5 ± 1.1	2.1 ± 1.0*	2.9 ± 1.5	2.5 ± 1.0	2.75 ± 1.36	2.42 ± 0.97*
Norepinephrine (µg/kg/minute)	0.22 ± 0.19	0.19 ± 0.19	0.20 ± 0.24	0.17 ± 0.19	0.21 ± 0.22	0.18 ± 0.19*
ScvO_2_ (%)	72 ± 9	75 ± 5	71 ± 9	73 ± 9	71 ± 9	74 ± 8
Pcv-aCO_2_ (mmHg)	5.7 ± 4.0	2.2 ± 2.4*	6.5 ± 4.2	8.5 ± 4.0	6.4 ± 4.1	7.6 ± 4.3

HR - heart rate; MAP - mean arterial pressure; CRT - capillary refill time; ScvO_2_ - central venous oxygen saturation; Pcv-aCO_2_ - central venous to arterial carbon dioxide difference.

* p < 0.05 post versus pre;

† p < 0.01 post versus pos. Results expressed as mean ± standard deviation and n normal/n abnormal.

Thirteen patients (31%) were renal responders (Table 2). There were no differences in any of the measured variables before the fluid challenge for the RR and non-RR, although creatinine levels were slightly higher for the RR (p = NS). MAP increased for RR (p = 0.051) in comparison to that in non-RR. Blood lactate levels decreased significantly for RR but not for non-RR. Pcv-aCO_2_ decreased in RR when compared to that in non-RR. Norepinephrine infusion rates decreased in the non-RR and RR groups, but with marginal significance for the RR group (p = 0.052).

A 10% increase in MAP (n = 17) and a decrease in HR (n = 4) and in the norepinephrine rate (n = 9) was not associated with reversal of oliguria. Even when considering a change in at least one of these three parameters, there was not an association with oliguria reversal.

**Figure 1:**
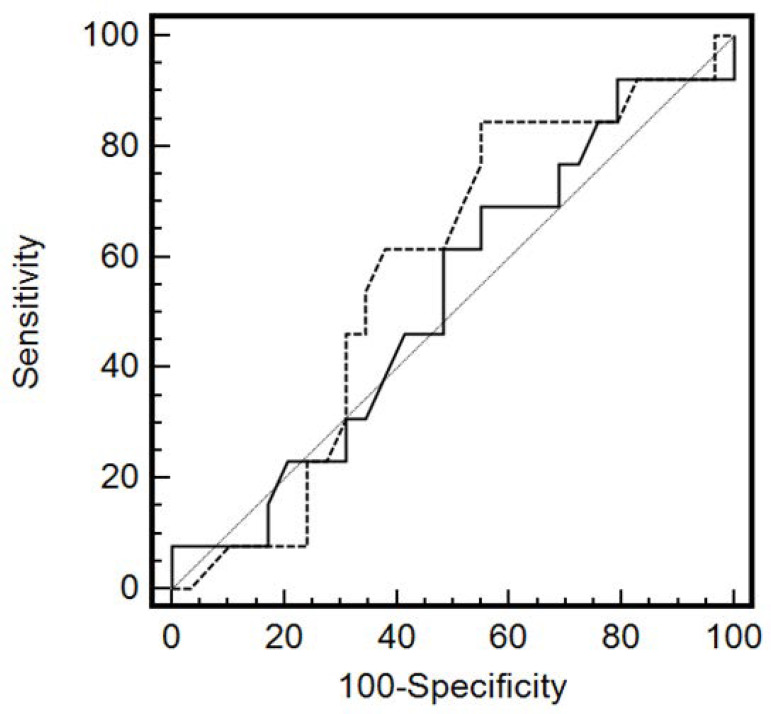
Receiver Operating Characteristic Curves for mean arterial pressure (dashed line) and heart rate (continuous line) at the time of oliguria recognition to predict oliguria reversal.

AUROC were calculated for MAP, HR, creatinine, urea, creatinine clearance, urea/creatinine ratio (available for all pts) and lactate (n = 37) before the fluid challenge. Table 3 and figure 1 shows the AUROC. None of these parameters were sensitive or specific enough to predict reversal of oliguria.

**Table 3:** Areas under the receiver operating characteristic curves to predict urine responsiveness

	Area (95%CI)	p value (Area = 0.5)
Mean arterial pressure (mmHg)	0.574 (0.412 - 0.725)	0.43
Heart rate (beats/minute)	0.525 (0.366 - 0.681)	0.80
Serum creatinine (mg/dL)	0.533 (0.373 - 0.688)	0.74
Blood urea (mmol/L)	0.592 (0.429 - 0.740)	0.35
Urea/creatinine ratio	0.525 (0.366 - 0.681)	0.79
Creatinine clearance	0.520 (0.361 - 0.676)	0.84
Arterial lactate (mmol/L)	0.540 (0.369 - 0.705)	0.69

95%CI - 95% confidence interval.

## DISCUSSION

In our study, most patients with transient oliguria did not increase their urine output after a fluid challenge. None of measured parameters were reliable predictors of urine responsiveness previous to fluid challenge.

In routine practice, low urine output often leads to performing fluid challenge in ICU patients. This is based on the hypothesis of a systemic hemodynamic contribution to low renal blood flow and low urine output.^(3)^ However, physiological reasons exist to consider oliguria a poor marker of hypovolemia or low intravascular volume.^(16)^ Shock, pain, and the perioperative period are associated with alterations in intrarenal hemodynamics and activation of the renin-angiotensin system, leading to antinatriuresis and antidiuresis.^(7,17)^ Our study shows that oliguria in normotensive ICU patients may not reflect hypovolemia in a large proportion of patients. Hence, fluid challenge may not translate into an increase in urine output and use of oliguria alone as triggers for fluid therapy is often not fully supported by physiological reasoning.^(6,12)^

The poor ability of systemic hemodynamic variables to predict an increase in urine output following fluid challenge was notable. First, RR and non-RR had similar baseline hemodynamic or perfusion markers. Second, although some parameters (blood lactate, norepinephrine rate, Pcv-aCO_2_, MAP) improved (p = NS) among RR, similar changes in the same parameters were not associated with reversal of oliguria and the prediction value of these variables was poor. These observations are consistent with previous investigations that showed the lack of correlation between systemic hemodynamic changes and response in urine output.^(4,5,18)^ In fact, even an increase in cardiac output after a fluid challenge is not consistently associated to a reversal of oliguria.^(4,12,13)^ Legrand et al.^(12)^ reported that the fluid challenge reversed oliguria in only one-half of the patients and that neither urinary sodium nor fractional excretion of sodium or urea were good predictors of the renal response to a fluid challenge.

The dissociation between systemic hemodynamics and urinary output suggests a predominant role of nonhemodynamic causes of decreased kidney function, particularly in sepsis.^(3)^ The underlying mechanisms that might be implicated are intrarenal shunting, inflammation with impaired microcirculation, mitochondrial dysfunction, and cell cycle arrest.^(2,19)^ Thus, fluids aimed to increase renal blood flow may not have the desired effect on the glomerular filtration rate if cardiac output is normal or increased. Our patients were hemodynamically stable, many not using or on low vasopressor dosing, and without obvious clinical or metabolic hypoperfusion. Thus, it is reasonable to speculate that most if not all had normal to increased systemic blood flow. Recently, the Andromeda study showed that less than 5% of patients were fluid responsive after early resuscitation. Although physicians in charge did not test fluid responsiveness to start a fluid challenge, patients were studied when they were stable and basically resuscitated, and it is reasonable to speculate that most of them were fluid nonresponsive.^(20)^

We superficially evaluated parameters that many clinicians consider markers of possible low intravascular volume that could justify a fluid challenge. Blood urea or mainly the urea/creatinine ratio were also poor predictors of reversal of oliguria. Although serum sodium concentrations were not available for most patients in our study, our results appeared to agree with others.^(12,13)^ Legrand et al. explored oliguria with low urine Na+ concentration in normotensive ICU patients and found that it may not reflect hypovolemia in a large proportion of patients.^(12)^ Urine sodium concentration is a biomarker of renin-angiotensin-aldosterone system activation that may be triggered by various factors. In a multicenter study, Pons et al. showed that urine biochemistry parameters, including fractional excretion of sodium and fractional excretion of urea, did not predict the rapid reversibility of AKI.^(21)^ Regulation of urine output is explained by many other factors, including tubular cell function and systemic inflammation, but are not explained solely by a decreased in urine or an increase in blood of these commonly used metabolic/electrolytes.^(3,22)^

Some studies emphasize that an improvement in renal hemodynamics seems to be essential for the increase in urine output to occur. Several investigators showed that changes in intrarenal vascular tone were correlated with changes in urine output and better predicted the increase in urine output after fluid administration than changes in systemic hemodynamics.^(18,21,23,24)^ An improvement in renal perfusion can be obtained and translated into an increase in urine output, even when there are no relevant changes in systemic hemodynamics. We did not measure renal hemodynamics due to the observational nature of our study, but reversal of oliguria was also obtained in patients without any systemic improvement. Although renal hemodynamics appear as an important parameter, none of these studies compared the effectiveness of changes in systemic and in intrarenal hemodynamics to predict changes in urine output after a fluid challenge.

It should be noted that the effects of fluid therapy are highly dependent on the phase of acute illness. Acutely, in a hypotensive and oliguric patient, the early effects of fluids in the resuscitation phase is not only likely beneficial but also potentially deleterious when established AKI later in the course of critical illness is almost the rule. Non-RR patients had a significantly lower arterial oxygen tension to inspired oxygen fraction (PaO_2_/SaO_2_) ratio and 62% were mechanically ventilated. Thus, it is reasonable to argue that this group of patients was sicker and suffered physiological disarrangements accompanying their critical illness.

We did not measure central venous pressure in our study or collected data on fluid balance. It is a limitation as a potential harmful effect of increased fluid balance has been suggested indicating an increased risk of AKI with increasing central venous pressure due to reduction of the transrenal pressure gradient for renal blood flow, while increasing interstitial and tubular pressure may reduce or abolish the net glomerular filtration pressure gradient.^(10,25,26)^ In addition, information on intrabdominal pressure was not available and intrabdominal hypertension causing intrarenal vascular congestion due to elevated renal vein pressure may induce AKI.^(27)^

Our study has additional limitations. First, the sample size was small and perfusion parameters were not available for all patients. Our patients had to be both oliguric and hemodynamically stabilized (normotensive), which may have limited the number of patients eligible for inclusion. Second, they had to be off drugs that induce diuresis, (e.g., diuretics). Third, monitoring of cardiac output was not performed to monitor stroke volume during the fluid challenge, and among RR one could argue that our data suggest a possible increase in blood flow not so evident because of the small sample. Fourth, data on urine obstruction or the use of nephrotoxins (drugs, radiologic contrast, and medications) were not recorded. In relation to this last limitation, someone could criticize the failure to include causes of nephrotoxicity as exclusion criteria, which would make the cohort less homogeneous. On the other hand, our focus was to show that the practice of challenging blood volume in oliguric patients is often ineffective and this approach is used even in patients with complex causes that lead to oliguria.

## CONCLUSION

In our study, oliguric patients, after achieving hemodynamic stability, did not increase urine output after a fluid challenge. Systemic hemodynamic or perfusion parameters were weak predictors of urine responsiveness. Our results suggest that volume replacement to correct oliguria in patients without obvious hypovolemia should be done with caution.
